# Editorial: A physiologically-based approach to study different types of locomotion in association with core performance

**DOI:** 10.3389/fphys.2024.1505881

**Published:** 2024-10-16

**Authors:** Erika Zemková, Magni Mohr, Tomáš Malý

**Affiliations:** ^1^ Department of Biological and Medical Sciences, Faculty of Physical Education and Sport, Comenius University in Bratislava, Bratislava, Slovakia; ^2^ Centre of Health Sciences, Faculty of Health, University of the Faroe Islands, Tórshavn, Faroe Islands; ^3^ Department of Sports Science and Clinical Biomechanics, SDU Sport and Health Sciences Cluster (SHSC), Faculty of Health Sciences, University of Southern Denmark, Odense, Denmark; ^4^ Faculty of Physical Education and Sport, Charles University in Prague, Prague, Czechia

**Keywords:** balance function, core stability/core strength, neurophysiological mechanisms, running, spine motion, walking

Good posture and strong core muscles are essential for most athletic movements (Kibler et al., 2006) but also for daily life activities (Hibbs et al., 2008). Among them, walking and running require lumbo-pelvic stability and mobility for efficient movement and high-level performance (Fredericson and Moore, 2005) as well as prevention of lower limb injuries (Leetun et al., 2004). This is especially important during extensive trunk motions while changing the direction of movement (Horníková and Zemková), an abrupt walk to run transition, or extreme uphill and downhill walking or running. Such repetitive trunk loading over time may contribute to occurence of back problems and lower limbs injuries. The main biomechanical risk factors leading to back problems in athletes are maladaptive spinal, spinopelvic and lower limb kinematics, side-to-side imbalances in axial strength and hip rotation range of motion, spinal overloading and deficits in movement pattern, whilst neurophysiological risk factors include neuromuscular imbalance, increased muscle fatigability, muscle dysfunction and impaired motor control (Zemková et al., 2023). Fatigue of the trunk muscles induced by excessive loading of the spine is one of the sources of back problems in athletes (Zemková et al., 2020). In particular, high training volume and repetitive motions are responsible for the high prevalence rates (Zemková et al., 2020). Lumbar muscle fatigue causes changes in the lumbar spinal curvature and this is functionally relevant in explaining the impaired ability to maintain balance after externally induced perturbations (Zemková et al., 2021). Core stability of the lumbopelvic hip complex can prevent buckling and help return to equilibrium after perturbation (Willson et al., 2005). On the other hand, reduced core stability can impair performance but also predispose to injury.

To avoid these unwanted effects requires a novel approach for studying the physiology of locomotion in association with the spine motion and balance function. This may provide a basis for designing the exercise programs specifically tailored for competitive athletes (Hibbs et al., 2008), healthy general population, as well as those suffering from movement disorders. Better neuromuscular control of postural and core stability contribute to more efficient functional movements specific to particular sports (Zemková and Zapletalová, 2022). Core stabilization and core strengthening exercises, alone or in combination with athlete training, contribute to increasing the performance, as well as reducing back pain in athletes (Zemková and Zapletalová, 2021). Specifically, ground-based free weight movements are effective for developing core strength and power due to the demands on force, velocity, and core stabilization that are similar to those of athletic skills (Willardson, 2007). Core strength has a significant effect on an athlete’s ability to generate and transfer forces to the limbs (Shinkle et al., 2012). This is also evidenced by the clear relationship between trunk muscle activity and lower extremity movement (Willson et al., 2005).

So far, much effort has been devoted to investigate biomechanical and physiological variations of locomotion, including walking, running, swimming or hopping. However, a surprising evidence gap is to what extent core strength contribute to effective locomotor performance and healthy back. Therefore, studying neurophysiological mechanisms underlying postural and core stability with special reference to locomotion is of great importance.

**FIGURE 1:**
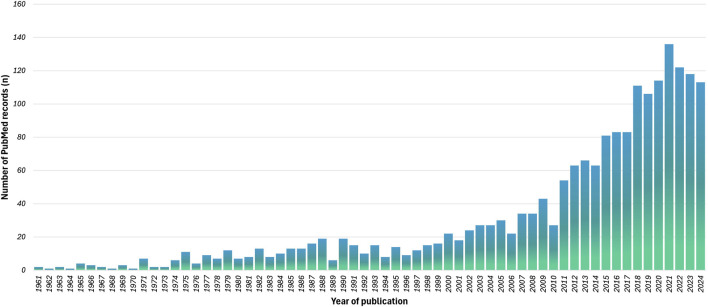
The results of a systematic search in PubMed according to the following Boolean search syntax: [“core performance” (All Fields)] OR [“core strength” (All Fields)] OR [“core stability” (All Fields)] AND [“locomotion” (All Fields)] OR [“walking” (All Fields)] OR [“running” (All Fields)] AND [“physiological mechanisms” (All Fields)] OR [“neurophysiological mechanisms” (All Fields)].

The fact that this issue is of great interest among researchers and practitioners is also evidenced by the exponentially increasing number of articles in the last decade. The Figure 1 illustrates the results of a systematic search in PubMed according to the Boolean search syntax: “core performance” OR “core strength” OR “core stability” AND “locomotion” OR “walking” OR “running” AND “physiological mechanisms” OR “neurophysiological mechanisms.” It includes 1747 items over 72 years.

Within this collection, 20 articles were submitted, of which 7 were rejected and 13 accepted. Actually, this Research Topic presents a collection of thirteen papers that exhibit substantial diversity in both content and methodological approaches.

Five of the studies focus on training interventions, employing various training methods. For instance, one study explores the effects of plyometric training on specific motor skills in young tennis players, while another examines the influence of horizontal plyometric training on volleyball-specific performance in post-peak-height-velocity female athletes. These studies offer valuable insights for coaches aiming to enhance athletic performance in youth populations. Another investigation tracks individual performance adaptations over 2 years of training in an elite 22-year-old aerobic gymnast, comparing outcomes between successive European Aerobics Championships, thereby providing detailed insights into performance variability at the elite level.

Two additional studies involve distinct populations—children and elderly women. The first is a 16-week school-based physical activity intervention targeting physical fitness in 8- to 9-year-olds, while the second assesses the effects of sensorimotor training on muscle strength and postural control in active elderly women aged 65–75 years. Both studies demonstrate significant intervention benefits, with potential implications for public health promotion.

Regarding training adaptations, another study employs a meta-analytic approach to evaluate the effects of blood flow restriction training on muscle activation and post-activation potentiation, illustrating how this method can effectively enhance lower extremity muscle engagement and post-activation performance.

The remaining papers address various performance dimensions across different sports and populations. One paper analyzes performance in young tennis players, highlighting motor skill differences across age categories, which could inform injury prevention strategies and training preparation. Another study explores several parameters of strength, morphological traits, and neuromuscular asymmetries in competitive soccer players, offering practical insights for trunk strength monitoring and development.

In relation to trunk strength, another study compares trunk rotational strength with shoulder rotational strength in athletes from mixed martial arts, tennis, swimming, and baseball. Both studies are critical for advancing knowledge on injury prevention strategies in sports.

Fatigue, a key focus area in sports physiology, is addressed in two studies: one investigates how fatigue impacts lower limb biomechanics during a forward lunge, while another evaluates the effect of molecular hydrogen supplementation on muscle performance, damage, and perceived soreness following two consecutive strenuous training sessions in elite fin swimmers, revealing potential benefits for muscle recovery.

Finally, two studies emphasize strength-related parameters. One compares the effects of various stimuli on maximal strength and power during bench press exercises, providing evidence for the most effective stimuli for improving both strength and power. The last study, a narrative review, synthesizes current research on the relationship between core strength and change of direction performance, highlighting the significance of core training for enhancing change of direction ability—a key performance indicator across many sports, making this area of interest highly relevant to performance physiology.

In the 2nd volume entitled “Neurophysiological basis of the relationship between core stability and human movement: Implications for sport and rehabilitation” we will continue to provide more information on this issue, focusing on the application of findings both in sports practice and clinical medicine. Then it is necessary to take into account the fact that research carried out in the rehabilitation settings cannot be applied to the sporting environment due to differing demands on the core musculature during sporting (dynamic movements, high loads) and everyday activities (slow movements, low loads) (Hibbs et al., 2008). Much evidence supports neurophysiological adaptations in body control induced by general conditioning exercises, however, little effort has been made to explain balance and locomotor adaptations induced by sport-specific exercises and their effects on athletic performance (Zemková and Kováčiková, 2023). While an enhancement in athletic performance is often attributed to an improvement of neuromuscular functions induced by sport-specific balance exercises, it can be equally well ascribed to their improvement by general body conditioning exercises. We believe that new contributions will address the complexity of this specific topic and present new insights into the knowledge and justification of research investigating the relationship between various movements and stability of the core musculature.
